# Acute appendicitis in young children less than 5 years: review article

**DOI:** 10.1186/s13052-017-0335-2

**Published:** 2017-01-26

**Authors:** Hamdi Hameed Almaramhy

**Affiliations:** Department of Surgery, College of Medicine, Taibah University, AL-Madinah Al-Munawarah, Kingdom of Saudi Arabia

**Keywords:** Acute appendicitis, Children, Under 5 years

## Abstract

Despite wide spread availability of sophisticated diagnostic imaging, acute appendicitis in pre-school children remains a diagnostic challenge. Most of these children present late, often with complications e.g. appendicular perforation, abscess formation and peritonitis and as result hospital stay is prolonged and is associated with increased morbidity and mortality.

The purpose of this article is to review peculiar features of acute appendicitis in preschool children.

## Background

Acute appendicitis is common surgical emergency among children (1–2% in pediatrics surgical admissions) [1–3]. Overall, 1–8% of children presenting with abdominal pain have acute appendicitis [4]. However, Appendicitis is uncommon in pre-school Children (2 to 9% children presenting with acute appendicitis) [5]. Despite the availability of advanced diagnostic imaging, the diagnosis of acute appendicitis in young children remains a challenge as most of such patients present late with complications e.g. perforation leading to abscess formation, generalized peritonitis and sepsis. The delay in the diagnosis of acute appendicitis has been attributed to nonspecific presentations, overlap of symptoms with many other common childhood illnesses, together with inability child to express and difficult abdominal examination in this age group. Misdiagnosis rate ranges from 28 to 57% in 2 to 12 year old children and approaches to nearly 100% in children younger than 2 years [6–8].

## Surgical anatomy

The vermiform appendix is a tube like diverticulum of the cecum with an average length of 4.5 cm in neonates and 9.5 cm in adults [9]. The base is wider and funnel shaped in neonates and infants with lesser chances of luminal obstruction. It takes the cylindrical adult shape at the age of 1 to 2 years.

The base of the vermiform appendix is less likely to be variable in position and lies on the posteromedial surface of the cecum at the convergence of its three taenia coli, while its tip is highly variable in position. The appendicular tip is retrocaecal in 28–68%, followed by pelvic position in 27–53%, subcaecal in 2%, anterior or preilleal in 1%, within hernial sac in 2%, right upper quadrant in 4%, and in left upper and left lower quadrants in less than 0.1% each [9].

Fetal and infantile appendices are generally freely mobile and less likely to be fixed with the cecum, ascending colon, to the posterior abdominal wall and there are greater chances of diffuse spillage of intestinal contents after the appendix perforates in such patients, compared to a localized abscess in elderly children. The variable tip and different positions of the vermiform appendix might explain the nonspecific presentations of acute appendicitis, e.g. In retrocaecal and sub serosal positions if the appendix gets inflamed the anterior abdominal pain and tenderness are less likely to develop. However, these patients usually experience more flank pain or back pain with longer duration of symptoms and with higher rates of perforation.

## Pathophysiology

The exact pathogenesis of acute appendicitis is multi factorial although it is still unclear. But it is irrefutable that obstruction of the lumen is the usually present. In preschool children this obstruction is usually due to lymphoid hyperplasia and less likely due to fecolith, as the appendix contains an excessive amount of lymphoid tissue in the submucosa which increase in size and number with growing age, reaching maximum in number and size during teenage with a higher possibility of developing acute appendicitis [1, 4]. Lymphoid hyperplasia is also associated with various inflammatory and infectious disorders such as gastroenteritis, amebiasis, respiratory infection, measles, and infectious mononucleosis. Faecoliths are formed by over layering of calcium salts and fecal debris on the inspissated feces within the lumen of the vermiform appendix. Luminal obstruction with continuous secretion and stagnation of fluids and mucus from epithelial cells result in increased intra-luminal pressure and distension of the appendix. Intestinal bacteria within the appendix multiply, and the edematous wall precipitates bacterial invasion. Also, the resulting compromise of the blood supply, decreased venous return, and eventually thrombosis of the appendicular artery and vein aggravates the inflammatory process, resulting in ischemia, necrosis, gangrene, and perforation.

The perforation of appendix result in either diffuse peritonitis, or localized appendicular abscess. Diffuse peritonitis is more common in younger children, due to a less developed omentum, whereas elderly children are relatively protected by well-developed omentum. The most common aerobic offenders for causing acute appendicitis are Escherichia coli, Klebsiella pneumoniae , peptostreptococcus, and pseudomonas species, and Bacteroides fragilis.

## Epidemiology

Acute appendicitis is one of the common causes of abdominal pain in children. The lifetime risk of developing acute appendicitis among males and females is 8.6 and 6.7%, respectively [1]. Although acute appendicitis is uncommon in infants and younger children, still neonatal as well as prenatal cases have been reported [10–18]. The incidence of acute appendicitis gradually increases after birth, peaks during the late teens and gradually declines in the geriatric age. Recently published studies have revealed that the incidence of acute appendicitis varies considerably according to sex, race, socioeconomic and immigrant status of the general population [19–22]. Its incidence has been reported to be declining in some western countries during recent years [23, 24]. During the late half of the 20th century, the incidence of appendectomy has been declining among children of various age groups. The incidence of acute appendicitis has declined from 3.6/10,000 to 1.1/10,000 among preschoolers, from 18.6/10,000 to 6.8/10,000 in children aged 5–9 years, and from 29.2/10,000 to 19.3/10,000 in children aged 10–14 years [25]. The decreasing incidence rates of acute appendicitis has been largely attributed to a better attention to various suggested etiological factors such as hygiene [26], diet [27], seasonal variation [28, 29], infection [4, 30], breast feeding [31] and genetic [32–34].

## Clinical presentation

During early childhood, presentation is atypical which makes the diagnosis more difficult. Moreover, the children of this age group have poor communication skills that can results in miss understanding of the disease process. The varied clinical presentation in different age groups is well explained by anatomical variation and pathophysiological differences responsible for acute appendicitis. These factors are of great concern to the clinicians and emphasize the need to properly investigating such patients in achieving a successful management protocol.

### Neonates (birth to 30 days)

In this age group, premature neonates are most likely to develop acute appendicitis [17, 35]. Here, luminal obstruction is not responsible for acute appendicitis. However, ischemia due to emboli or thrombotic event, obstructed internal or external hernia, cardiac anomalies and distal colonic obstruction as in Hirschprung’s disease, are the more likely causes of neonatal acute appendicitis.

Pain and nausea cannot be well appreciated as an evidence of acute appendicitis in these neonates. These patients usually present with abdominal distension in 60% to 90%, vomiting 59%, palpable mass 20–40%, irritability or lethargy in 22% and 12–16% with cellulitis of abdominal wall. However, hypotension, hypothermia, right hip stiffness and respiratory distress have been observed in some cases as well [12, 36–39].

### Infants and toddlers (less than 3 years)

The prominent symptoms in this age group are vomiting (85% to 90%), pain (35 to 81%), fever (40–60%), and diarrhea (18 to 46%). Other common symptoms during this age group are irritability (35% to 40%), cough or rhinitis (40%), grunting respiration (8% to 23%), right hip mobility restriction, pain and limping in 3% to 23%. Vomiting and irritability are also presenting symptoms of many other disorders at this age like gastroenteritis, mesenteric adenitis, intussusception, otitis media, and upper respiratory tract infections. On physical examination, majority of the infants (87% to100%) have temperature higher than 37oc and diffuse abdominal tenderness (55% to 92%); whereas localized right lower quadrant tenderness is observed in less than 50% of cases. Other noticeable signs are lethargy (40%), abdominal distension (30–52%), rigidity (23%), and abdominal or rectal mass (30%) [40–42]. As the presentation of acute appendicitis in this age group is nonspecific, vague, the mean time interval between the onsets of symptoms and final diagnosis is usually 3 to 4 days. This delay in diagnosis most often results in perforation (82–92%), and bowel obstruction 82% [40–42].

### Preschool (age 3–5 years)

Acute appendicitis is still rare up to 6 years of age, accounting for only less than 5% of all childhood appendicitis [4, 43]. With growing age, children are able to communicate well and can describe the symptoms of acute appendicitis, early diagnosis of acute appendicitis becomes more easy and accurate. The majority of children in this age group present with complex complaints of 2 days duration and up to 17% have the symptoms for more than 6 days before the final diagnosis is reached [4]. In this age group, abdominal pain is the most common presenting symptom (89% to100%), followed by vomiting (66% to100%), fever (80% to 87%) and anorexia (53% to 60%). On examination, localized right lower quadrant tenderness (58% to 85%) predominates over the diffuse tenderness (19% to 28%). Other physical signs include involuntary guarding (85%), rebound tenderness (50%), and temperature greater than 37.5 o c (82%) [4].

## Reasons for the misdiagnosis and higher incidence of perforation

The non-specific clinical presentation in children less than 5 years, as well as difficult communicate with them , inadequate physical examination, irritability, and overlap of symptoms with other common childhood illnesses attribute to delayed diagnosis of acute appendicitis and high misdiagnosis rate. Hence they are more likely to develop complications such as perforation and abscess formation other factors contribute to perforation are thin-walled appendix, and inadequate omental barrier. The differential diagnosis in these children include, but not limited to, acute gastroenteritis, upper and lower respiratory tract infections, urinary tract infections, cholecystitis, constipation, intussusception , pelvic inflammatory disease, blunt abdominal trauma, obstructed hernia, testicular torsion, orchitis, nephrolithiasis, right hip septic arthritis, dehydration, sepsis, encephalopathy, and meningitis.

The overall rate of missed diagnosis ranges from 70 to100% among children of 3 years and younger, 19 to 57% in preschool age group (with perforation in 43% to 72% of the cases). This rate decreases to 12 to 28% for school age children, reaching less than 15% in adolescents [6, 43, 44].

In a clinical study, up to 15% of patients were seen twice or more in the emergency department before the diagnosis of acute appendicitis was made and the common features for misdiagnosed patients were relatively short duration of symptoms at the initial visit, most of them attended late at night, had fewer physical findings on examination, and were not well investigated [44]. The rate of misdiagnosis rises as age decreases, and young children have a 5-fold risk of complicated appendicitis [45]. In a study on 102 children where investigators explored risk factors for appendicular perforation, it was found that the duration of pain and the presence of appendicolith were the most statistically significant factors [46].

## Investigations

**Fig. 1:**
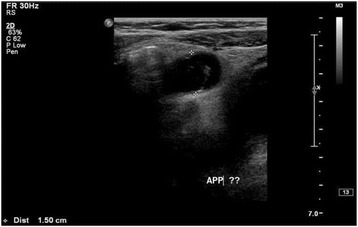
Ultrasound abdomen for 5 years old boy presented with abdominal pain showed an 1.5 cm non compressible tubular-like structure suggestive of appendicitis

**Fig. 2:**
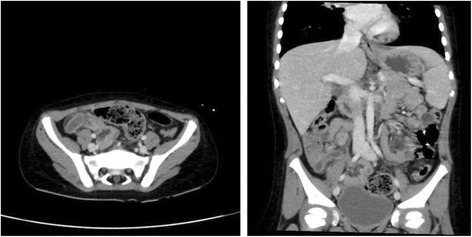
Cross section and coronal view CT scan for the same patient shows prove the diagnosis of acute appendicitis

The diagnosis of acute appendicitis is not easy in young children. It necessitates the need for certain laboratory and radiological investigations in all age groups for making an accurate diagnosis,:

A. Laboratory Evaluation:
  i. Biological markers:
    Various biochemical and hematological markers have been established for improving the diagnostic accuracy of acute appendicitis in younger children (Table 1). Below the common ones are discussed
    - Complete blood count (CBC ) and CRP:
      Worldwide, CBC is the most commonly advised laboratory investigation in children with suspected acute appendicitis. Although the white blood cell (WBC) count is increased in acute appendicitis, still it is non-specific and insensitive. WBC count is also elevated in other disease processes such as gastroenteritis, mesenteric lymphadenitis, pelvic inflammatory disease and certain other infections. Furthermore, the WBC count cannot differentiate between a complicated and an uncomplicated acute appendicitis. Elevated neutrophil count along with the total WBC count further helps in the diagnosis of acute appendicitis. The sensitivity and specificity of leukocytic count to diagnose acute appendicitis varies from 60 to 87%, to 53–100% in different published international studies [43, 47–52]. In situations of high susceptibility of acute appendicitis, elevated WBC count further enhances the accuracy of clinical diagnosis, while a normal count of WBC cannot exclude the diagnosis [53–55]. However, in cases of less chances of acute appendicitis, a high WBC count warrants further radiological evaluation and clinical observation.
      C-reactive protein (CRP) is a nonspecific inflammatory mediator. It has a sensitivity of 43% to 92% and a specificity of 33% to 95% for diagnosing acute appendicitis in children presenting with abdominal pain. However, it is more sensitive than WBC count in diagnosing appendicular perforation and abscess formation, which are more common in children. The sensitivity of leukocytosis and increased neutrophil count may approach 98% with an elevated CRP for diagnosing acute appendicitis [50, 56].
    - Neutrophils to lymphocytes (N/L) ratio and the mean platelets volume (MPV):
      It has been suggested that neutrophils to lymphocytes ratio and the mean platelets volume may be used as markers to decrease the rate of negative appendectomy. Goodman et al., and Yazic et al., have found that N/L ratio of more than 3.5 is a sensitive indicator for the diagnosis of acute appendicitis [57, 58]. Albayrak et al., found a statistically significant reduction in MPV in their case-control study on adult appendicitis comparing acute appendicitis cases with a healthy control group [59]. The same findings have been reported in similar study designs in pediatric population by Vijay et al. [60], and Bilici et al. [61], although another study contradicted these findings [62].
  ii. Urine Analysis:
    Urine analysis is advised to rule out urinary tract infection. However 7–25% of pediatric patients with acute appendicitis have more than 5 WBCs or RBCs per high power field in the urine sample [63, 64]. Chen et al., in a newly published article, reported diagnostic value of acute appendicitis by urine analysis [65]. He concluded that positive urinary ketone bodies and nitrates might be the important markers that help in diagnosing a perforated acute appendicitis.
    The decision to advise WBC count, neutrophil count, and CRP, or urine analysis is usually based on the clinical impression, duration of symptoms, and the preference of the emergency room physician or consultant surgeon.
B. Imaging evaluation:
  i. Plain x-ray Abdomen:
    Plain abdominal radiographs are routinely performed in case of acute abdomen. Radiographic findings, suggestive of acute appendicitis are right sided scoliosis, soft tissue mass, localized ileus, bowel obstruction, free peritoneal fluid, and faecolith. The most specific among these findings for diagnosis of acute appendicitis is faecolith found in 28 to 33% of patients with inflamed appendix and exists in less than 1 to 2% of cases without inflammation of appendix. Interestingly, perforation was found to be present in 45 to 100% of cases where x-rays revealed a calcified appendicolith [66, 67]. Most of the recent studies predict that normal plain radiographs in acute appendicitis are misleading in the majority of cases. Therefore, plain abdominal radiographs are mostly recommended in those cases of acute abdomen, where intestinal obstruction, peritonitis, renal or gallstones are suspected [68].
  ii. Ultrasonography (USG):
    The ultrasound findings suggestive of acute appendicitis are: distension and obstruction of the appendiceal lumen, swollen appendix (diameter > 6 mm)-Fig. 1, an appendicolith , a target sign with five concentric layers , high echogenicity surrounding the appendix, , pericecal and perivesical free fluid, and thickened bowel loops with deceased peristalsis [69]. Its sensitivity and specificity range from 80 to 92% and 86 to 98%, respectively [70–73]. In meta-analysis of 26 studies evaluating the role of ultrasound in diagnosis of AP in 9356, the pooled sensitivity were 88%( 95% CI = 86–90), and specificities were 94% ( CI = 92–95) [74]. The rate of visualization of an inflamed appendix varies from 22 to 98% [75]. The American College of Radiology recommended that a child with atypical or equivocal clinical presentation of acute appendicitis, and non-visualization or non-diagnostic findings on ultrasonographic examination (USG) should be observed with serial physical examinations and repeated imaging, which can result in marked reduction of CT scan imaging in children [76–79].
  iii. Computed Tomography Scan (CT scan) and MRI:
    CT scan has been extensively used when the ultrasound failed to identify the inflamed appendix. The diagnostic criteria on CT scan-Fig. 2-; include swollen appendix (diameter more than 6 mm), fat streaking , focal caecal apical thickening, lymphadenopathy, presence of an appendicolith , abscesses, cut off of colonic contrast at the proximal appendiceal lumen ( arrowhead sign), and separation of contrast in the caecal lumen from a proximal appendicolith ( caecal bar). Various studies have reported sensitivity of CT scan in the diagnosis of appendicitis between 87 and 100%, and a specificity of 83% to 100% [80–82]. It is useful in reducing the number of negative appendectomies, and is helpful for making an alternate diagnosis for abdominal pain. In an observational study of 125 children by CT scan imaging for suspected acute appendicitis, 62 of them were found to have another diagnosis such as mesenteric adenitis, inflammatory bowel disease, and ovarian cyst [83]. Currently, no significant data is available on the sensitivity and specificity of CT scan for the detection of acute appendicitis in young children. But, In a multi-center study of 55,227 child it was found the use of pre-operative CT scan in children less than 5 years of age, significantly reduced the negative appendectomy rate ( NAR) when compared to those not utilize this facility [84]. However, it is very crucial to understand that the ionizing radiations emitting from CT scan has been shown to be associated with a higher lifetime risk of cancer in children [85–87]. MRI has not been routinely used in diagnosis of acute appendicitis. In one study, non-blinded radiologists detected all cases of non-perforated appendicitis that were diagnosed on ultrasound [88]. Large scale research work is required to establish its sensitivity and specificity in the diagnosis of inflamed appendix.
C. Other investigations
  Barium enema, radioactive-tagged leukocyte scans, and diagnostic laparoscopy have recently been used in the diagnosis of acute appendicitis in children but their diagnostic accuracy is not yet established.
D. The role of a scoring system in the diagnosis of acute appendicitis:
  Several scoring systems have been designed as an alternative or complementary to improve the diagnostic accuracy of acute appendicitis [89–95]. An improved diagnostic accuracy in adult population has been reported in some studies. Alvarado scoring system (MANTRELS) is one of commonly used scoring systems (Table 2).
  In this scoring system, patients with a score less than 5 can be investigated for non-appendicular cause of pain, those with score of 5–6 should be admitted for observation and further investigations, while patients with a score of 7 or above are most likely positive for acute appendicitis and need surgery. The Alvarado score of 7 or higher has a sensitivity of 88% to 90% and a specificity of 72% to 81% for acute appendicitis [96–98]. In 2002, Samuel first time published another scoring system specific to acute appendicitis in children (Table 3) [99].
  Samuel described that the Pediatric Appendicitis Score (PAS) was more reliable and precise to differentiate children suffering from acute appendicitis. However, various other prospective cohort validation studies failed to establish the diagnostic accuracy described by Samuel [96, 100, 101]. Furthermore, the literatures failed to reveal the significant advantage in children less than 4 years, as this scoring system is better applicable for children who can communicate well about the shifting of pain and anorexia. Others argue that they help for better clinical decision and in decreasing perforation rate [89, 101–104]. Although multiple published studies validated the PAS, only one study has enrolled children younger than 4 years of age [100]. Recently, Salo et al. [105], evaluated the accuracy of PAS in a retrospective study among 122 child aged 1 to 14 years who underwent appendectomy for suspected appendicitis. The cohort was gathered into two age groups: ≥4 years (102 child) and <4 years (20 child). They found that the mean PAS was lower among the younger age group (5.3 vs 6.6) with statistical significance (*P* = 0.005), despite that younger children had more severe appendicitis (75.0 and 33.3% respectively, *P* = 0.001). The study concluded that PAS had low sensitivity in both groups, with a significantly lower sensitivity among the younger children. In recent prospective study on 311 patients comparing the sensitivity and specificity of Alvarado and pediatric appendicitis scoring (PAS) systems in diagnosing acute appendicitis in children, Pogorelic et al. found no significant difference between both systems [106].

**Table 1:** The most common biological markers that have been studied in diagnosis of appendicitis

In blood/serum	In urine
White blood cell count (WBC)	Urine 5-hydroxyindoleacetic acid (5-HIAA)
Differential leukocyte counts (DLC	Urine leucine-rich alpha glycoprotein-2 (LRG)
C-reactive proteins (CRP)	
Erythrocyte sedimentation reaction (ESR)	
Tumor necrosis factor alpha (TNF-alpha), acid)	
Alpha1-glycoprotein (alpha1gp)	
Leucocyte elastase complex (elastase)	
Interleukin-8 (IL-8)	
Interleukin-6 (IL-6)	
Interleukin-10 ( IL-10)	
Granulocyte colony stimulating factor	
Interferon gamma	
Soluble intercellular adhesion molecule-1	
Matrix metalloproteinase-9	
Tissue inhibitor metalloproteinase-1	
Serum amyloid A	
Plasma calprotectin	
Plasma serotonin	
Serum leucine-rich alpha glycoprotein-2 (LRG)	
Procalcitonin

**Table 2:** MANTRELS (Alvarado) Score, reference No. 89

Features	Points
Migration of pain from central abdomen to right lower quadrant	1
Anorexia	1
Nausea with vomiting	1
Tenderness in right lower quadrant	2
Rebound tenderness	1
Elevated temperature ≥38 °c (100.4 °F)	1
Leukocytosis (≥10, 400/mm3)	2
Shifted WBC count ( 75% neutrophils)	1
Total possible points	10

**Table 3:** Pediatric Appendicitis Score, reference No. 99

Features	Points
Migration of pain	1
Anorexia	1
Nausea/vomiting	1
Right lower quadrant tenderness	2
Cough/hopping/percussion tenderness in the right lower quadrant	2
Elevated temperature (>38-C)	1
Leukocytes Q10.000/KL > 10,000	1
Polymorphonuclear neutrophilia >75%	1
Total points	10

## Management

Children diagnosed with acute appendicitis should be immediately admitted for observation and/or emergent appendectomy. Children with atypical presentation require surgical consultation. A protocol using the appropriate application of scoring system, radiological adjuncts and in-patient close clinical observation will help to diagnose or exclude acute appendicitis. In-patient observation by a surgeon can help in differentiating atypical presentation of acute appendicitis from other disorders. A group of patients with very low risk based on the Alvorado or PAS scoring system can be discharged from the emergency room with an advice of repeat evaluation after 8 to 12 h [107, 108].

A protocol of active monitoring that involves frequent clinical examinations every 4 to 6 h, with or without repeat ultrasound, for patients without evidence of obvious physical signs mandating surgical exploration (ie, the presence of rebound tenderness or peritonitis) will enhance the diagnostic yield and will decrease the utilization of CT scan and radiation risk [3, 109].

Historically, open appendectomy has been practiced in young children all over the world for acute appendicitis. However with the advent of minimally invasive techniques, laparoscopic appendectomy has become increasingly popular among pediatric surgeons. Recently, researchers have started the use of antibiotics alone to treat low grade appendicitis as an alternative to surgery when the family refuses or prefers to avoid surgery [110]. Traditionally, appendicular mass in very young age group has been managed as in adult population by conservative management, followed by interval appendectomy with good outcome, although this group of children poorly respond to conservative management. It is widely accepted that patients with appendicular abscess can be managed with immediate CT scan, or ultrasound guided per-cutaneous drainage and parenteral broad spectrum antibiotics, followed by interval appendectomy [111, 112]. The patients can later get the benefits of minimally invasive approach as well [113]. In case of failed per-cutaneous drainage, open or laparoscopic surgical drainage is an alternative. However, we should bear in mind that young children don’t form localized abscess as older children and early intervention is recommend in such patients. Both mortality and morbidity rates in acute appendicitis have been significantly reduced with early diagnosis, broad spectrum antibiotics, fluid resuscitation, better anesthesia, well equipped intensive care units and improved surgical skills [114, 115].

## Conclusion

Acute appendicitis in young children of preschool age group and infants is uncommon. Delay in the diagnosis and management predominantly result from poor communication skill, failure to elicit physical signs in irritable children, atypical presentation, and overlap of symptoms with other disorders. Late presentation leads to onset of complications such as appendicular perforation and peritonitis. Diagnosis in this age group requires a high index of suspicion, a careful history, and serial physical examinations. Admit and observe policy is highly recommended in equivocal cases. Diagnostic accuracy can be enhanced with the appropriate use of imaging tools such as ultrasound and CT scan depending upon the available facilities. Early diagnosis and prompt surgical intervention can reduce the morbidity and mortality rates associated with complicated appendicitis.

## Abbreviations

AP: Acute appendicitis
CBC: Complete blood count
CI: Cephalic index
CRP: C-reactive protein
CT scan: Computed tomography scan
MPV: Mean platelets volume
MRI: Magnetic resonance imaging
NAR: Negative appendectomy rate
PAS: Pediatric Appendicitis Score
USG: Ultrasonography
WBC: White blood cell
